# *Mycobacterium bovis* BCG Surface Antigens Expressed under the Granuloma-Like Conditions as Potential Inducers of the Protective Immunity

**DOI:** 10.1155/2019/9167271

**Published:** 2019-06-03

**Authors:** Yingyu Chen, Lia Danelishvili, Sasha J. Rose, Luiz E. Bermudez

**Affiliations:** ^1^Department of Biomedical Sciences, College of Veterinary Medicine, Oregon State University, Corvallis, OR, USA; ^2^College of Veterinary Medicine, Huazhong Agricultural University, Corvallis, OR, USA; ^3^The National Key Laboratory of Agricultural Microbiology, Huazhong Agricultural University, Wuhan, China; ^4^Department of Microbiology, College of Science, Oregon State University, Corvallis, OR, USA

## Abstract

Bovine tuberculosis (bTB) is a highly transmissible infection and remains of great concern as a zoonosis. The worldwide incidence of bTB is in rise, creating potential reservoir and increased infection risk for humans and animals. In attempts to identify novel surface antigens of *Mycobacterium bovis* as a proof-of-concept for potential inducers of protective immunity, we investigated surface proteome of *M. bovis* BCG strain that was cultured under the granuloma-like condition. We also demonstrated that the pathogen exposed to the biologically relevant environment has greater binding and invasion abilities to host cells than those of bacteria incubated under regular laboratory conditions. A total of 957 surface-exposed proteins were identified for BCG cultured under laboratory condition, whereas 1,097 proteins were expressed under the granuloma-like condition. The overexpression of Mb1524, Mb01_03198, Mb1595_p3681 (PhoU1 same as phoY1_1), and Mb1595_p0530 (HbhA) surface proteins in *Mycobacterium smegmatis* leads to increased binding and invasion to mucosal cells. We also examined the immunogenicity of purified recombinant proteins and tested *M. smegmatis* overexpressing these surface antigens for the induction of protective immunity in mice. Significantly high levels of specific IgA and IgG antibodies were observed in recombinant protein immunized groups by both inhalation and intraperitoneal (IP) routes, but only IP delivery induced high total IgA and IgG levels. We did not detect major differences in antibody levels in the *M. smegmatis* group that overexpressed surface antigens. In addition, the bacterial load was significantly reduced in the lungs of mice immunized with the combination of inhaled recombinant proteins. Our findings suggest that the activation of the mucosal immunity can lead to increased ability to confer protection upon *M. bovis* BCG infection.

## 1. Introduction

Bovine tuberculosis (bTB) is a significant zoonotic threat that is associated with both animal losses with substantial economic consequences and a high risk for human infection. The global impact of bTB is estimated at US $3 billion per year [1]. Although a number of measures have been adopted to control the infection, the incidence of the disease is on the rise [2]. The diagnoses of bovine tuberculosis in many countries still depend on indirect methods, such as using PPD, and in the majority of the cases, chemotherapy is not a practical alternative to treat infected animals. Respiratory and gastrointestinal tracts are the main routes for acquiring *Mycobacterium bovis*. If inhaled, bacteria are phagocytosed by macrophages or bind and invade mucosal epithelial cells [3–5]. The initial interaction may play an important role in determining the progression of the disease. Vaccination, therefore, remains the most effective and feasible approach to control the infection. Although some countries still utilize the human *M. tuberculosis* vaccine (*Mycobacterium bovis* BCG) to immunize cattle, current bTB epidemiologic studies indicate that it does not protect animals from infection. In fact, many studies in Mexico, Ethiopia, and New Zealand have demonstrated that vaccination of cattle with BCG is associated with short-lived protection, and the level of protection varies from animal to animal [6–8]. In addition, the BCG immunization with multiple-doses for bTB shows poor protection and does not improve outcome, or in some cases, it even worsens the outcome [9].

*M. bovis*, the main agent causing bTB, is an intracellular bacterium, and it is generally agreed that the cellular-mediated immunity plays a pivotal role in the host control of the infection [10]. Although there is debatable evidence about the protective role of the humoral immunity against *M. tuberculosis* infection [11], antibody response, which has been ignored as a component of the protection against bTB for many years, has more recently shown to play a role in host response against *M. tuberculosis* [12]. In addition to significant cell-mediated immunity, antibodies that were generated against specific mycobacterial surface antigens can activate essential protective responses against intracellular mycobacterial pathogens [13]. Studies in cattle have determined that antibodies against *M. bovis* antigens are commonly observed, although no clear correlation with protection has been established [14–17].

The infection with *M. bovis* mainly affects the respiratory tract; however, infection through the gastrointestinal tract in both humans and animals is not uncommon. In most cases, the pathogen can be transmitted from an infected animal to a naïve host by aerosol. Once inhaled, *M. bovis* readily establishes infection in the alveolar space of the lung [18, 19]. Although macrophages are considered as primary host cells for *M. tuberculosis* infection, the fact is that the number of alveolar epithelial cells outnumbers the macrophages by severalfold in the alveolus space and the chance that *M. bovis* will encounter the alveolar epithelial cells is significantly greater than the binding to alveolar macrophages [3]. The alveolar epithelial cells are the critical first physiological barrier to prevent *M. bovis* from entering the bloodstream, and recent work has shown that lung mucosal cells play a significant role in the pathogenesis and immunity against tuberculosis infection [20, 21].

The mucosal vaccination with *M. bovis* BCG has been demonstrated in a challenge using the natural route of infection [22]. A study by Moliva and colleagues, evaluating whether incubation of *M. bovis* BCG to airway secretion would induce specific immune response in mice, showed that alveolar lining fluid enhances *M. bovis* BCG vaccine efficacy against *M. tuberculosis* in a CD8^+^ T cell-dependent manner [23]. Despite these findings, T cell-mediated responses have been investigated for many years and have shown that the vaccination with BCG leads to the generation of weak effector memory T cells and tissue resident memory cells, and it lacks the mucosal expression of chemotactic receptors [24].

The mucosal vaccine against *M. tuberculosis* in humans had been recently investigated and was demonstrated to exhibit promising efficacy to prevent the infection [25, 26]. The mucosal vaccination of animals against *M. bovis* infection has also been tested. Many studies have reported the response to nasal and endobronchial vaccination with a variety of response [11, 14]. Because of the high genome similarity between *M. bovis* and *M. tuberculosis* [27], we hypothesized that a mucosal vaccine against *M. bovis* can be also developed.

Our previous work revealed that under different environmental conditions mimicking the environment of the granuloma, *M. tuberculosis* has different binding and invasion ability and suggests that the pathogen acquires a different phenotype when in the granuloma [28].

In this study, we investigated (i) if exposure to granuloma-like conditions would influence *M. bovis* ability to bind and invade the host cells, (ii) if the granuloma-like condition effect the composition of surface proteins when compared with the regular laboratory condition, (iii) if overexpression of surface antigens would further promote the binding and invasion ability of *M. bovis*, and (iv) if bacterial surface antigens can induce mucosal immunity *in vivo*. Due to the cost limitation and requirement of a BSL-3 animal facility for *M. bovis* challenge, we decided to perform a proof-of-concept experiment using *M. bovis* BCG strain.

## 2. Materials and Methods

### 2.1. Bacteria

*Mycobacterium bovis* BCG Pasteur strain was kindly provided by Brigitte Gicquel (Institut Pasteur, Paris, France). *Mycobacterium smegmatis* mc^2^ 155 was kindly provided by Jacobs, Jr. (Albert Einstein School of Medicine, Bronx, NY). Mycobacterial strains were cultured on Middlebrook 7H10 agar supplemented with 10% oleic acid, albumin, dextrose, and catalase to log phase (Hardy Diagnostics, Santa Maria, CA). Prior to the assays, BCG Pasteur strain was resuspended in 2 ml of Hanks' balanced salt solution (HBSS) and passed through a 23-gauge needle 10 times. After 10 min rest, the top 1 ml was adjusted to McFarland standard 2 (approximately 3 × 10^8^ cells/ml) and used as the inoculum. The inoculum was observed under the microscope to ensure the dispersal of the sample. In addition, the serial dilutions were plated for CFU reads to calculate exact concentration of the inoculum. Viability of *M. bovis* BCG was also determined by the LIVE-DEAD assay (Invitrogen).

### 2.2. Cell Culture

A549 human type II alveolar epithelial cells, MDBK bovine epithelial cells, and THP-1 phagocytic cells were purchased from ATCC. A549 cells and MDBK cells were maintained in DMEM supplemented with 10% heat-inactivated fetal bovine serum. A total of 10^5^ cells were added to each well of a 24-well tissue culture plate. Human mononuclear phagocyte THP-1 cells were cultured in RPMI-1640 medium supplemented with 10% heat-inactivated fetal bovine serum. THP-1 cells were treated with 20 ng/ml phorbol 12-myristate 13-acetate (PMA, Sigma) and seeded in tissue culture plates for 24 hrs to allow for maturation. Next day, monolayers were replenished with fresh RPMI-1640 medium and rested for an additional 48 hrs. Cells were seeded at 90% confluence.

### 2.3. Environmental Conditions

BCG was incubated in different conditions: (1) regular laboratory culture condition: 37°C, 20% O_2_, and pH 7.2; (2) granuloma-like condition: increased osmolarity in 7H9 broth supplemented with 0.3 M dextrose and no oxygen. Oxygen deficient condition was obtained in an anaerobic jar (VWR) with pH 6.0 at 37°C. After 24 hrs of incubation, bacteria were washed at 4°C, resuspended in HBSS, passed through the 23-gauge needle 10 times, and used for further assays.

### 2.4. Invasion and Binding Assay

A549 cells and MDBK cells were infected with live BCG at a multiplicity of infection (MOI) of 10 bacteria to 1 host cell for 30 min and 1 h and either incubated at 4°C for the binding (no invasion) or 37°C for the invasion assay as previously described [3]. MOI of 1 and 0.1 has been also tested, and MOI of 10 was chosen to be used in the assays. The cell monolayers were gently washed three times with PBS, and then for staining of the intracellular bacteria, they were permeabilized with 0.1% Triton X-100 for 15 min. In addition, monolayers were lysed in water for 30 minutes, and the cell lysates were serially diluted and plated onto 7H10 agar plates. Bacterial CFUs were quantified after three weeks of incubation. Binding and invasion of *M. smegmatis* to A549 and MDBK cells were carried out as described for *M. bovis* BCG.

### 2.5. Biotin Labeling and Purification of BCG Surface Proteins

*M. bovis* BCG culture from different conditions were resuspended in 1 mg of Sulfo-NHS-LC-biotin (Pierce, Rockford, IL) reconstituted in 1 ml HBSS. Bacteria were biotin labeled for 30 min at 4°C with gentle agitation. The unbound Sulfo-NHS-LC-biotin was inactivated and removed by washing bacteria three times with 10 mM glycine in HBSS. Labeled bacteria were resuspended in the guanidinium lysis buffer (10 mM EDTA, 2 Mm EGTA, 0.1% Tween-20, 6 mM guanidinium-HCl; in PBS, pH 7.2) and disrupted by bead-milling machine using 100 *μ*m glass beads (Sigma, St. Louis, MO). Samples were cleared by centrifugation at 15,000 rpm for 5 min. The supernatants were then processed for isolation of biotinylated proteins out of the total protein sample by affinity purification with magnetic streptavidin-coated C1 Dynabeads (Invitrogen). Briefly, aliquots of 1.5 ml of protein sample were incubated for 1 h at 23°C with 80 *μ*l of C1 Dynabeads and microcentrifuged. The beads were washed twice with the guanidinium lysis buffer followed by the washing with 0.05% Tween-20 in PBS (pH 7.2). Beads were resuspended in 35 *μ*l of 1% SDS supplemented with 10 mM EDTA in H_2_O and incubated at 65°C for 10 min. 45 *μ*l of 2× Laemmli buffer was added to each sample, boiled for 10 min, and then cleared by centrifugation at 2,000 rpm for 10 min. Biotinylated proteins were visualized by SDS-PAGE, as previously published [29].

### 2.6. Mass Spectrometry and Data Analysis

Isolated surface proteins of *M. bovis* BCG were further processed for proteomic analysis at the Mass Spectrometry Facility of Oregon State University. Briefly, concentrated proteins were processed using the ProteaseMax trypsin digestion (Promega), following the manufacturer protocol. Digested proteins were further purified with a Michron Peptide CapTrap column and C18 column (Agilent Zorbax). Samples were sequenced and analyzed in the Mass Spectrometry Facility at Oregon State University using a LC-MS/MS with a Thermo LTQ-TF MS coupled to a Waters nanoAcquity UPLC system. The data obtained were analyzed against the Proteome Discoverer and Mascot database as previously described [29].

### 2.7. Overexpression of *M. bovis* BCG Proteins in *M. smegmatis*

*M. bovis* BCG selected genes were amplified using FideliTaq PCR Master Mix (Affymetrix, Santa Clara, CA) using the primers listed in Table 1. The 6xHis tag was incorporated in primers. The PCR-generated fragments were cloned into the mycobacterial shuttle vector pMV261 containing HSP60 promoter and kanamycin resistant marker. The resultant plasmids were propagated in *Escherichia coli* DH10 B and then electroporated into *M. smegmatis* mc^2^ 155. Transformants were selected on 50 *μ*g/ml of kanamycin containing Middlebrook 7H10 agar plates and screened by PCR for kanamycin gene as previously described [30]. The protein expressions in *M. smegmatis* were confirmed by western blotting using the anti-HIS probe antibody.

### 2.8. Expression and Purification of *M. bovis* BCG Recombinant Proteins

*M. bovis* BCG selected genes were amplified using FideliTaq PCR Master Mix (Affymetrix, Santa Clara, CA) using the primers listed in Table 2. The PCR-generated fragments were cloned into a pET6xHN-N vector encoding ampicillin resistance. The resultant plasmids were transformed into Bl21 (DE3) cells, and transformant colonies were selected on LB agar plates containing 100 *μ*g/ml of ampicillin. The protein expression in *E. coli* was confirmed by western blotting using the anti-HIS probe antibody. Briefly, samples of 150 mg of bacteria were harvested and resuspended in 700 *μ*l 0.5%SDS with 0.5% protease inhibitor cocktail (Sigma). Bacteria were mechanically disrupted in a bead-milling machine with 0.1 mm silica beads (6 cycles, 20 s each @ max speed). Lysates were centrifuged at 12,000 g for 5 min, and supernatants were mixed with Laemmli buffer (+5% BME). The remaining pellets were also processed and resuspended in 200 *μ*l denaturing buffer (500 mM NaCl, 7 M urea, 20 mM TriseHCl, 10 mM imidazole) and Laemmli buffer (+5% BME). Both soluble and nonsoluble samples were boiled for 10 min, run on 12% Mini-PROTEAN precast SDS-PAGE gel (Biorad, Hercules, CA), and transferred to a nitrocellulose membrane. After 1 h exposure with the blocking buffer (4% BSA with PBS-Tween) at room temperature, the membranes were further incubated with anti-HIS monoclonal antibody for 1 h followed by incubation with IRDye-680 streptavidin (Licor, Lincoln, NE) according to the manufacturer protocol. The biotinylation patterns were visualized on an Odyssey Scanner (Licor).

### 2.9. Vaccination and Challenge of Mice

The *in vivo* study was approved by the IACUC under the protocol number 4880. TiterMax® Gold Adjuvant was purchased from Sigma-Aldrich.

A total of 6 groups (9 mice in each group) of the six-week-old female C57BL/6 mice were used in this study. The equal amounts of the purified recombinant proteins were mixed together at a total concentration of 50 *μ*g. At this concentration, 100 *μ*l was used for intraperitoneal injections (chosen due to concern that the preparation would be painful if delivered into the muscle or subcutaneously) and 20 *μ*l for inhalation. *M. smegmatis* clones overexpressing the target *M. bovis* BCG proteins were mixed together, and a total amount of 10^7^ were used for inhalation. Boost was carried out 1.5 weeks after primary immunization.

The groups with 9 mice in each group were set as follows:

1. Intraperitoneal immunization using 50 *μ*g purified mixed recombinant proteins with the TiterMax® Gold Adjuvant
2. Intraperitoneal injection with PBS in the TiterMax® Gold Adjuvant as a control for adjuvant effects
3. Inhalation with 50 *μ*g purified mixed recombinant proteins by using micropipettes and delivering 20 *μ*L into the nostril
4. Inhalation with PBS
5. Inhalation with 10^7^ *M. smegmatis* overexpressing the target *M. bovis* BCG proteins
6. Inhalation with 10^7^ *M. smegmatis* with pMV261 vector as a control

Three weeks after vaccination, all mice were intranasally challenged with 10^7^ BCG Pasteur strain preexposed to the granuloma-like conditions. For the infection, mice were briefly anesthetized with isoflurane and a micropipette was used to introduce the bacterial inoculum into the airway [31, 32]. Two weeks later, antisera were collected for IgG detection and lungs for IgA detection. One mouse in each group was euthanized for histological section, and others were euthanized to determine the bacterial load. The lungs were homogenized, debris removed by filtration, and serially diluted samples were plated on the Middlebrook 7H10 agar plates for the CFU counts of bacilli. The spleens were harvested, homogenized, serially diluted, and plated onto 7H10 agar plates as described for the lungs. The number of viable bacteria was counted after three weeks of incubation at 37°C.

The timing for the experiments was chosen based on the principle that mucosal antibodies and effective innate immune response would be present at 2-3 weeks after immunization, and therefore, the timing would be enough to examine the level of infection. This timing has been used in many other challenges [5, 18].

### 2.10. Mice Infection with Different Phenotypes of *M. bovis* BCG

C57BL/6 black mice (total of 24 mice) were infected intravenously either with the wild-type *M. bovis* BCG cultured in 7H9 Middlebrook broth, termed as a regular lab condition, or with bacteria cultured under granuloma-like conditions as previously described [33]. Infection was carried out with 3 × 10^6^ bacteria. After 3 days (baseline infection) and two weeks after infection, six mice were harvested from the control and experimental groups to determine the initial tissue load (lungs and spleens). Organs were obtained and homogenized as previously described [33]. The serially diluted homogenates were plated on 7H10 agar supplemented with carbenicillin, amphotericin B, trimethoprim-sulfamethoxazole, and polymyxin B and incubated for 20 days to determine bacterial CFUs. The differences in bacterial CFU per gram of tissue were compared between control and experimental groups.

### 2.11. IgG Detection

The total levels of IgG were detected using the commercially available kit (eBioscience, Vienna, Austria) according to the manufacturer's protocol. Briefly, 96-well plates were coated with 100 *μ*L/well of capture antibody (pretitrated, purified anti-mouse IgG monoclonal antibody) in PBS buffer overnight at 4°C. The wells were washed two times with PBS and blocked overnight at 4°C. Wells were incubated with 10 *μ*L diluted (1 : 100) sera and 50 *μ*L diluted detection antibody (pretitrated, HRP-conjugated anti-mouse IgG polyclonal antibody) for 2 hrs followed by three washes with PBS. The substrate solution (tetramethylbenzidine) was added for 15 min, and reaction was stopped with stop solution of 2N·H_2_SO_4_. Plates were recorded at 450 nm wavelength. The standard curve was made to quantify the total IgG levels. Five *μ*g/ml mixed *M. bovis* BCG recombinant proteins were used to coat the 96-well plate to detect the specific IgG followed by the same steps as described for total IgG. No standard was used for the specific IgG.

### 2.12. IgA Detection

The total levels of IgA were detected using the commercial kit (eBioscience, Vienna, Austria) according to the manufacturer's protocol. Briefly, 96-well plates were coated with 100 *μ*L/well of capture antibody (pretitrated, purified anti-mouse IgA monoclonal antibody) in PBS buffer overnight at 4°C. The wells were washed two times with PBS and blocked overnight at 4°C. Wells were incubated with 10 *μ*L diluted (1 : 50) lung homogenates and 50 *μ*L diluted detection antibody (pretitrated, HRP-conjugated anti-mouse IgA polyclonal antibody) for 3 hrs at room temperature followed by four washes with PBS. Substrate solution was added for 15 min, and reaction was stopped with 2N·H_2_SO_4_. Plates were recorded at 450 nm wavelength. The standard curve was created to quantify the total IgA levels.

Five *μ*g/ml mixed *M. bovis* BCG recombinant proteins were used to coat the 96-well plate to detect the specific IgA followed by the same steps as described for total IgA. Lung homogenates were 20 times diluted for specific IgA detection. No standard was used for specific IgA.

### 2.13. Statistical Analysis

*In vitro* experiments were repeated at least three times, and data shown are means of the replicates and standard error. Student's *t*-test was used for analysis of side-by-side comparisons, and variance between experimental groups was assessed by one-way ANOVA. A value of *P* < 0.05 was considered to be significant. Graph Pad Prism software version 5.0 was used for statistical analysis.

## 3. Results

### 3.1. Granuloma-Like Environment Enhances *M. bovis* BCG Binding and Invasion of Lung Epithelial A549 and THP-1 Macrophage-Like Cells

**Figure 1:**
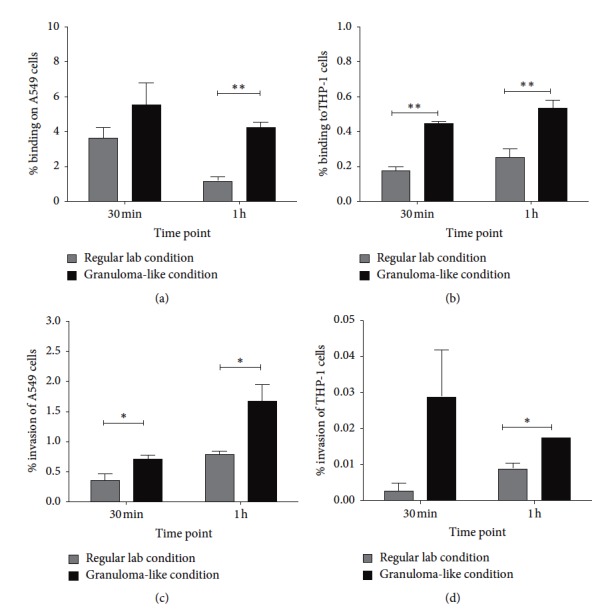
Binding and invasion assays of *M. bovis* BCG exposed to granuloma-like (pH 6.0, 0.3 M dextrose, anaerobic) and regular lab (pH 7.2, 20% O_2_) conditions. (a) Percentage of bacterial binding to A549 lung epithelial cells. (b) Percentage of bacterial binding to THP-1 macrophages. (c) Percentage of bacterial invasion of A549 cells. (d) Percentage of bacterial invasion of THP-1 cells. Results represent mean ± standard error of three independent experiments. ^*∗*^ *P* < 0.05, and ^*∗∗*^ *P* < 0.01. Percentage of binding and invasion is calculated from the total *M. bovis* BCG inoculum added to cell monolayers at 0 time point.

*M. bovis* BCG Pasteur strain was exposed to two different pH (6.0 and 7.2), hyperosmolarity (0 and 0.3 M dextrose), and anaerobic or aerobic conditions. Figures 1(a) and 1(b) demonstrate that bacteria bind efficiency at 4°C when exposed to A549 epithelial cell and THP-1 macrophages for 30 min and 1 h. *M. bovis* BCG exposed to the environment mimicking the granuloma-like (pH 6.0, 0.3 M dextrose, anaerobic) condition for 24 hrs is able to bind to A549 cells with significantly greater efficiency than bacteria cultured in regular laboratory conditions (pH 7.2, 20% O_2_) (Figure 1(a)). Likewise, *M. bovis* BCG exposed to granuloma-like condition attaches with significantly higher efficacy to THP-1 macrophages when compared to the binding percentage of bacteria cultured in regular laboratory conditions at both 30 min and 1 h time points (Figure 1(b)).

In addition, invasion assay was carried out to address the question whether different environmental conditions could influence *M. bovis* uptake by lung epithelial cells and macrophages. Figures 1(c) and 1(d) demonstrate bacterial invasion efficiency at 37°C when exposed to A549 epithelial cell and THP-1 macrophages for 30 min and 1 h. *M. bovis* BCG exposed to granuloma-like condition was able to invade epithelial cells as well as THP-1 macrophages with significantly greater efficiency than bacteria cultured in regular laboratory conditions after 1 h exposure to host cells. Differences in *M. bovis* BCG uptake were seen at 30 min after infection as well as in both cell lines.

### 3.2. Proteomic Analysis of the Surface of *M. bovis* BCG Exposed to the Biologically Relevant Condition

**Figure 2:**
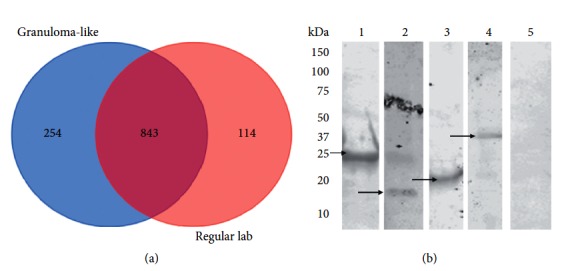
*M. bovis* BCG surface proteins expressed in different conditions. (a) Venn diagram showing the number of overlapping and unique set of bacterial surface proteins synthesized in granuloma-like and regular lab conditions. (b) Western blotting of *M. smegmatis* cell lysate overexpressing Mb1524, Mb01_03198, PhoU1, and hbhA proteins using anti-His monoclonal antibody. Lane 1, pMV261- PhoU1; Lane 2, pMV261-Mb01_03198; Lane 3, pMV261-hbhA; Lane 4, pMV261-Mb1524; Lane 5, pMV261 control. Arrows indicate expressed proteins.

Due to the fact that *M. bovis* BCG exposure with the granuloma-like condition induced efficient binding and invasion to the host cells, in attempts to identify surface molecules contribution to these phenotypes, we profiled and identified changes on the surface proteome of *M. bovis* BCG expressing the regular laboratory and granuloma-like condition phenotypes. *M. bovis* BCG surface-exposed proteins were selectively labeled and extracted for mass spectrometry analysis. A total of 1,211 proteins were detected by mass spectrometry in both samples (supplemental material (available here)). *M. bovis* BCG cultured in the regular laboratory condition identified 957 proteins, whereas the granuloma-like condition had 1,097 proteins. The Venn diagram (Figure 2(a)) shows that 114 proteins detected in *M. bovis* BCG cultured in the regular lab condition were absented in granuloma-like condition, while 254 proteins were uniquely expressed in *M. bovis* BCG of granuloma-like condition. Twelve proteins were selected for further studies based on their antigenic properties; however, we were able to successfully isolate four *M. bovis* BCG recombinant proteins and express them in *M. smegmatis*.

### 3.3. *M. smegmatis* Clones Overexpressing *M. bovis* BCG Surface Proteins Efficiently Bind and Invade Host Cells

**Figure 3:**
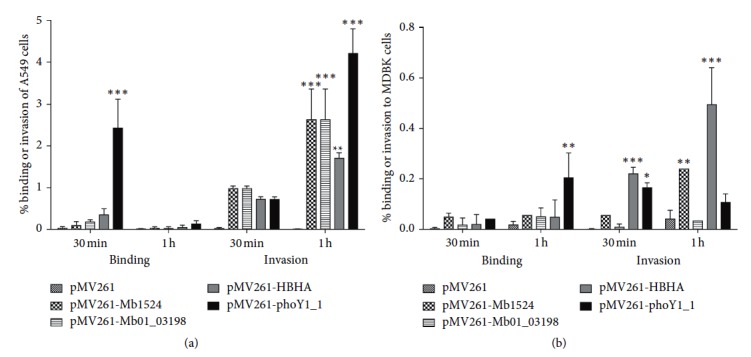
Binding and invasion assays of (a) A549 cells and (b) MDBK cells using *M. smegmatis* overexpressing pMV261-Mb1524, pMV261-Mb01_03198, pMV261-PhoU1, and pMV261-hbhA constructs. Results represent mean ± standard error of three independent experiments. ^*∗*^ *P* < 0.05; ^*∗∗*^ *P* < 0.01; ^*∗∗∗*^ *P* < 0.0001 compare with the control pMV261 construct of *M. smegmatis*.

*M. smegmatis* is fast growing nonpathogenic mycobacteria that poorly binds and invades the host epithelial cells but, at the same time, it is efficiently phagocytized by macrophages. In order to determine if selected *M. bovis* BCG proteins contribute to the host cell binding and invasion, we constructed *M. smegmatis* clones expressing all twelve *M. bovis* BCG surface-exposed proteins. Using the western blotting, it was found that *M. bovis* Mb1524, Mb01_03198, Mb1595_p3681 (PhoU1), and Mb1595_p0530 (HbhA) proteins were successfully expressed in *M. smegmatis* mc^2^ 155 (Figure 2(b)). The overexpressed clones, alongside with *M. smegmatis* control containing just the pMV261 vector, were tested for the greater ability to bind and invade A549 epithelial and MDBK bovine epithelial cells. While *M. smegmatis* overexpression of the PhoU1 protein had significantly higher binding ability when compared with all three other clones at 30 min after infection (Figure 3(a)), all four clones were able to invade A549 cells with greater efficiency than control *M. smegmatis* containing the pMV261 vector at 1 h of the infection. Only *M. smegmatis* PhoU1 clone showed significantly greater binding ability to MDBK cells at 1 h compared with the control. The invasion experiments demonstrate that the clones expressing hbhA and PhoU1 proteins had significantly higher percentage than other clones at 30 min after infection. *M. smegmatis* overexpressing the Mb1524 protein invaded better only at 1 h (Figure 3(b)).

### 3.4. Phenotypes of *M. bovis* BCG Infection in Lungs and Spleens of Mice

As shown in Table 3, infection of C57BL/6 mice with 7H9 broth-grown (regular lab condition) and granuloma-like phenotypes showed significantly different growth rate in the lungs and spleens of mice at two weeks after infection. *M. bovis* BCG exposed to granuloma-like condition displayed more virulent phenotype, and bacterial load in infected animals was over one-log higher when compared to the same organism but grown under the regular condition at two weeks after infection (Table 3).

### 3.5. *M. bovis* Recombinant Proteins Induce Protective Immunity in Mice Immunized by Inhalation and IP Routes

**Figure 4:**
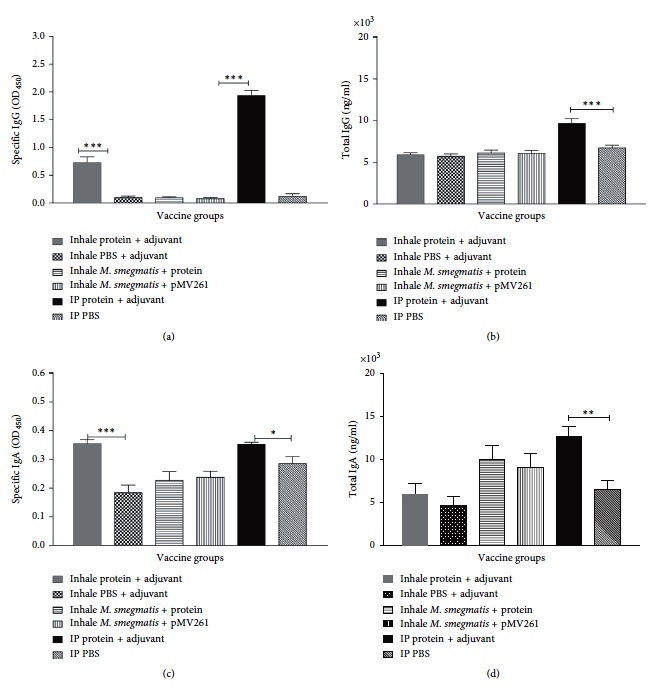
Specific and total antibody responses in C57BL/6 mice (*n* = 9/group) immunized either intranasally or intraperitoneally with the mixture of *M. bovis* four recombinant surface antigens or with M. smegmatis clones overexpressing these antigens for a total of two times at weeks 0 and 1.5. All experimental groups of mice were intranasally challenged with 107 BCG preexposed to the granuloma-like conditions, and two weeks later, antisera were collected for IgG and lungs for IgA detection by ELISA. The results for specific IgG (a) and IgA (b) are presented as OD450, whereas total IgG (c) and IgA (d) levels are calculated in ng/ml for the individual mice, and the mean values for each group are provided. ^*∗*^ *P* < 0.05; ^*∗∗*^ *P* < 0.01; ^*∗∗∗*^ *P* < 0.0001.

The mice were immunized intranasally with the mixture of *M. bovis* Mb1524, Mb01_03198, PhoU1, hbhA recombinant proteins, and the TiterMax® Gold Adjuvant. The phosphate buffered saline with adjuvant was used to immunize the control group of mice. The third group of the mice was administered by intraperitoneal injection with the mix of *M. smegmatis* clones overexpressing *M. bovis* surface proteins and tested if this immunization could trigger protective immunity. *M. smegmatis* clone with empty pMV261 vector served as a control. Serums were collected from all mice, and IgG levels were determined. It was found that only recombinant protein immunized groups by both, inhalation and intraperitoneal, routes had significantly high levels of specific IgG antibodies synthesized in the blood when compared with the control groups (Figure 4(a)). The significant greater total level of IgG was identified in the recombinant protein immunized group by IP injection (Figure 4(b)), but no significant changes were observed in other experimental groups when compared to the control group.

To analyze mucosal antibody responses in the airway, IgA levels were measured as well. Our results indicate that production of IgA had similar trend as IgG. The recombinant protein immunized group induced significantly high levels of the specific IgA antibody delivered via inhaling or IP routs, but no significant changes were observed in other experimental groups when compared with the control groups (Figure 4(c)). The significant production of the total IgA antibody was seen only in the experimental group of mice immunized with the recombinant proteins via IP (Figure 4(d)).

### 3.6. Bacterial Loads in the Lungs of the Preimmunized and BCG Challenge Groups

**Figure 5:**
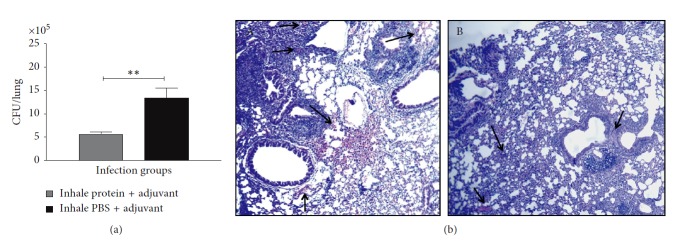
The number of viable bacteria in the lungs of the BCG challenged mice. (a) The colony forming units of *M. bovis* BCG were determined in the lungs of control nonimmunized (PBS inhalation) and the recombinant protein mix immunized mice (*n* = 9/group) that were challenged with *M. bovis* BCG as described in Section 2. Mice were sacrificed two weeks after the bacterial inoculation to identify the viable BCG. Results are expressed as CFUs for the individual mice, and the mean values for each group are provided. ^*∗*^ *P* < 0.05 (Student's t-test). ^*∗∗*^ *P* < 0.01. (b) The histopathology of lungs of mice challenged with *M. bovis* BCG after inhalation of PBS (A) or recombinant protein (B) immunization.

To measure the protective efficacy of immunization, we determined the bacterial load in the lungs of the experimental and control groups of mice during the intranasal BCG challenge. Our results demonstrate that the bacterial colony-forming units (CFUs) were significantly reduced in the lungs of the inhaled mice group that was preimmunized with the mix of recombinant proteins when compared with the inhaled PBS plus adjuvant control group (Figure 5). Histopathological observation also indicated that the level of infection was significantly reduced as shown in Figure 5(b), with fewer bacteria and significant less inflammatory response in mice preexposed to the mixture of proteins than in mice infected without local vaccine stimulation. In a similar fashion, mice exposed to *M. smegmatis* and a mixture of surface proteins controlled the infection more effectively than mice that did not received *M. bovis* surface proteins by aerosol. Not surprisingly, IP immunization appears to be ineffective in inducing protection.

## 4. Discussion

In the active tuberculosis cavity, *M. bovis* resides within the environment that is completely different than the one found in laboratory conditions and, therefore, the phenotype expressed by the pathogen within a biologically relevant environment likely differs from the phenotype that bacteria acquires in the culture growth medium. In this study, we aimed to identify proteomic changes on the bacterial surface during exposure to granuloma-like condition and characterize some of these proteins that may have a role in the bacterial pathogenesis and/or contribute to the development of specific host immune responses.

One of the possibilities for limited protection of BCG as a vaccine is that the whole bacteria may express immune suppressive molecules, which may attenuate the host immune responses, making it less effective. By using the surface proteins that can influence the initial interaction between the pathogen and the host, most likely we may prevent the existing suppressive tendency. The data reported in this study support that *M. bovis* BCG cultured in granuloma-like condition can bind and invade more efficiency to A549 epithelial cells, THP-1 macrophages, and MDBK epithelial cells than bacteria exposed to the regular laboratory condition and may efficiently escape the defense mechanism presented on the mucosal surface.

Subsequent studies in mice showed similar results where the *M. bovis* BCG strain grown in granuloma conditions expressed more virulent phenotype than the strain grown in the regular laboratory condition. Because of the fact that exposure to granuloma-like condition enhanced bacterial binding and uptake into the host cells, we focused on identification of *M. bovis* BCG surface antigens that may have a role in the invasion-enhanced phenotype. In addition, because these antigens are exposed on the surface of bacteria, they could also trigger an adaptive antibody response and prevent future infection by a similar route. A comparative proteomics approach was used to identify *M. bovis* BCG surface proteins unique to the granuloma-like condition and to the regular laboratory condition, and then selected surface proteins were tested for increased binding and invasion assays. By comparing the data obtained from two observations, we were able to decide on which proteins to choose for further characterization. At first, we selected proteins that have been shown to have a role in the initial infection of virulent mycobacteria and the host respiratory mucosa, such as HBHA and PhoU1. Secondly, two additional proteins selected were involved in triggering of signal transduction in the host cell and lipid transport in the bacterium. Proteins Mb1524, Mb01_03198, and PhoU1 were differentially expressed in *M. bovis* BCG cultured in granuloma-like condition. The protein hbhA contributes to bacterial interaction with epithelial cells and also plays a role in extrapulmonary dissemination during tuberculosis infection [31, 34]. In addition, hbhA protein was highly expressed in *M. bovis* BCG of “granuloma-like conditions,” supporting the selection of this protein for inclusion in the immunizing cocktail.

We found that *M. bovis* BCG selected surface proteins of the granuloma-like condition were related to the binding and invasion of the host cells and also induced mucosal immune response in mice. The hypothetical Mb1524 gene encodes a protein containing the conserved domain of the regulator of protease activity HflC, stomatin/prohibitin superfamily, whose function remains unknown; however, it was proposed that through this domain, proteins could interact with the cell membrane and initiate signaling [35]. We also selected the hypothetical Mb01_03198 protein for further characterization. The domain search for Mb01_03198 identified the diacylglycerol kinase family enzyme motif involved in the lipid transport and metabolism and domains of transcriptional regulator XRE-family helix-turn-helix and transcription elongation factor GreA/GreB, C-term.

The PhoU1 gene is ubiquitously present in virtually every bacterial species, including *M. tuberculosis*. The PhoU1 encodes the phosphate-specific transport system accessory protein PhoU involved in regulation of phosphate uptake. The evidence suggests that two putative orthologs of PhoU, PhoY1 and PhoY2, promote *M. tuberculosis* persistence phenotype by Pst-mediated phosphate sensing and enhance increased resistance to antibiotics both *in vitro* and in mice [36, 37]. In the study addressing the *M. tuberculosis* response to several stress conditions, PhoU was found to have a major role in maintaining metabolic homeostasis and adaptation to stress conditions [38], also offering possible explanation for PhoU related drug tolerance. Here, we demonstrate that phoY1_1 overexpression on the surface of *M. smegmatis* helps bacteria to efficiently bind and invade both epithelia cells and MDBK cells when compared with the wild-type *M. smegmatis* control.

The heparin-binding hemagglutinin (hbhA) is a well-studied virulence factor of *M. tuberculosis*, *M. avium*, and *M. leprae* and plays a crucial role in bacterial binding to epithelial cells and to other nonphagocytic cells [31, 34, 39]. It has been demonstrated that hbhA is involved in extrapulmonary dissemination during tuberculosis infection and in the binding of *M. leprae* to the respiratory mucosa [31, 34, 40]. The mucosal immunization with the yeast-expressed recombinant hbhA impairs extrapulmonary dissemination of *M. bovis* BCG and, in combination with a mucosal adjuvant, hbhA induces immune protection against mycobacterial infection by triggering the systemic and local humoral immunity [13]. Although hbhA was present on *M. bovis*, BCG grown in both phenotypes of granuloma-like and regular laboratory conditions, the potential function and the effect of the protein on the immune response were chosen as selection criteria. We further confirmed the function of hbhA in binding and invasion of human epithelial cells and bovine MDBK cells.

Recent studies have used BCG vaccination given by different routes, for example, orally to immunize badgers with evidence of some degree of protection [41]. The main reason that we focused on investigation of the antibody response was to evaluate whether protection can be achieved by interfering the initial interaction between the host and bacteria. Mb1524, Mb01_03198, phoY1_1, and hbhA were further investigated *in vivo* for their antigenicity. Our results indicate that while *M. smegmatis* clones overexpressing BCG proteins were not all associated with improvement in the immune antibody response by either IgA or IgG, the delivery of mixture of recombinant proteins by both intranasal immunization or intraperitoneal injection routes stimulated specific IgA and IgG antibody levels and synthesis of total IgA and IgG. It is experimentally proven that increased levels of IgA and IgG influence mucosal immune response and protect the host from *M. bovis* infection [10, 32, 42]. In this study, we demonstrate that the increase in specific IgA is associated with significant changes in bacterial burden in the lungs of mice receiving recombinant protein mixture by the intranasal immunization.

## 5. Conclusions

Bovine tuberculosis is a great concern for animal health, and as a zoonosis, it has serious implications in human health. The bTB is highly transmissible disease both by respiratory and gastrointestinal rotes. In attempts to identify phenotypes acquired by the pathogen in the host lung, bacterial surface proteins were identified under the granuloma-like condition and selected proteins were used to immunize mice. Immunization was followed by challenge with *M. bovis* BCG. The combination of four *M. bovis* proteins had significant protection against challenge when compared with the nonimmunized mice group. Our results have direct implications in designing of mucosal vaccination strategies for bTB and improving a protective efficacy at the mucosal surface against *M. bovis* BCG by using recombinant protein mixture of Mb1524, Mb01_03198, PhoU1, and hbhA. Our results also highlight the importance of the mucosal vaccination against bovine tuberculosis using novel and biologically relevant antigens as vaccine candidates. The addition of other surface proteins in the vaccine cocktail preparation may improve the protection by inducing opsonizing IgA. It is known that toxic exposure to antigens many times leads to superior immune response than systemic immunization [10, 42]. One possible limitation of the current study is that mice were challenged with *M. bovis* BCG, an attenuated strain compared with the wild-type *M. bovis*. Nonetheless, our initial intent was to establish a proof-of-concept. As demonstrated through experiments reported in this study, the use of surface antigens that are expressed in the pathogen of “granuloma-like condition” is most likely crucial for achieving the optimal immune-related protection in the mucosa and to prevent the establishment of the infection.

## Figures and Tables

**Table 1:** Primers used for construction of *M. smegmatis* clones overexpressing *M. bovis* BCG surface antigens in the pMV261 vector. His-tag was incorporated in the primer.

Genes	Primers
Mb1524	5′-TTTTTGAATTCCATCATCATCATCATCATCAAGGAGCCGTTGCT-3′ 5′-TTTTTGTCGACCTATTGAGTCAACCTGGGGGG-3′
Mb01_03198	5′-TTTTTGAATTCCATCATCATCATCATCATGACACAACTGTC-3′ 5′-TTTTTGTCGACCTAAGCGTCGATCCC-3′
PhoU1	5′-TTTTTGAATTCCATCATCATCATCATCATCGGACGGTCTAT-3′ 5′-TTTTTGTCGACTCAGTAAGTGGAAATCTCGTCCT-3′
HBHA	5′-TTTTTGAATTCCATCATCATCATCATCATGCTGAAAACTCGAAC-3′ 5′-TTTTTGTCGACCTACTTCTGGGTGACCTTCTT-3′

**Table 2:** Primers used for construction of recombinant *M. bovis* BCG surface antigens in the pET6xHN_N vector of *E. coli*.

Genes	Primers
Mb1524	5′-TTTTTGTCGACCAAGGAGCCGTTGCT-3′ 5′-TTTTTGAATTCCTATTGAGTCAACCTGGGGGG-3′
Mb01_03198	5′-TTTTTGTCGACGACACAACTGTCGCT-3′ 5′-TTTTTGAATTCCTAAGCGTCGATCCC-3′
PhoU1	5′-TTTTTGTCGACCGGACGGTCTAT-3′ 5′-TTTTTTGAATTCCAGTAAGTGGAAATCTCGTCCT-3′
HBHA	5′-TTTTTGTCGACGCTGAAAACTCGAAC-3′ 5′-TTTTTGAATTCCTACTTCTGGGTGACCTTCTT-3′

**Table 3:** Infection of C57BL/6 mice with *M. bovis* BCG grown under laboratory and granuloma-like conditions.

Infection/condition	CFU/g^a^			
	3 days		2 weeks	
	Lung	Spleen	Lung	Spleen
*M. bovis* BCG (regular lab)	1.6 ± 0.4 × 10^6^	8.4 ± 0.4 × 10^4^	1.4 ± 0.6 × 10^4^	7.0 ± 0.5 × 10^2^
*M. bovis* BCG (granuloma-like)	2.2 ± 0.3 × 10^6^	7.6 ± 0.2 × 10^4^	1.7 ± 0.7 × 10^5*∗*^	5.1 ± 0.4 × 10^3*∗*^

^a^12 mice/group infected with 3 × 10^6^ bacteria. ^*∗*^The significance of differences between the granuloma-like and regular lab conditions, recorded with bacterial CFU at 2 weeks of infection, was *P* < 0.05.

## Data Availability

The raw data and mutants are available upon request.

## Supplementary Materials

Supplementary Materials BCG surface proteins isolated from bacteria growing in granuloma-like conditions. The proteins are organized by the number of peptides sequenced.
